# Novel specialized guidewire for bridging deployment into the right hepatic duct via endoscopic ultrasound-guided hepaticogastrostomy for malignant hilar biliary obstruction

**DOI:** 10.1055/a-2440-7314

**Published:** 2024-11-08

**Authors:** Tadahisa Inoue, Rena Kitano, Tomoya Kitada, Kazumasa Sakamoto, Satoshi Kimoto, Jun Arai, Kiyoaki Ito

**Affiliations:** 1 12703 Department of Gastroenterology, Aichi Medical University, Nagakute, Japan

Endoscopic ultrasound (EUS)-guided biliary drainage is a useful salvage technique when endoscopic retrograde cholangiopancreatography (ERCP) fails. However, achieving bilateral drainage under EUS guidance presents significant challenges, particularly in cases of malignant hilar biliary obstruction 1 . One reported method involves bridging the right hepatic duct combined with EUS-guided hepaticogastrostomy (EUS-HGS) 2 . Despite the use of this approach, navigating a guidewire can be particularly difficult when the bifurcation angle between the right and left hepatic ducts is acute.

To address this limitation, we proposed a novel guidewire with a specialized design. Compared to conventional guidewires, this 0.025-in wire features a notably larger angled tip (60°) and a longer tip length from the bend to the end (14 mm) ( Fig. 1 ). Additionally, the core wire in the tip is passively flexible, which prevents traction loss and slipping toward the common bile duct upon initial contact with the right hepatic duct. These features enable selective access, even in cases of sharp bifurcations. The shaft is designed with a textured surface that reduces friction with other instruments, ensuring excellent torqueability and rotational performance, which further improves its selectivity.

**Fig. 1:**
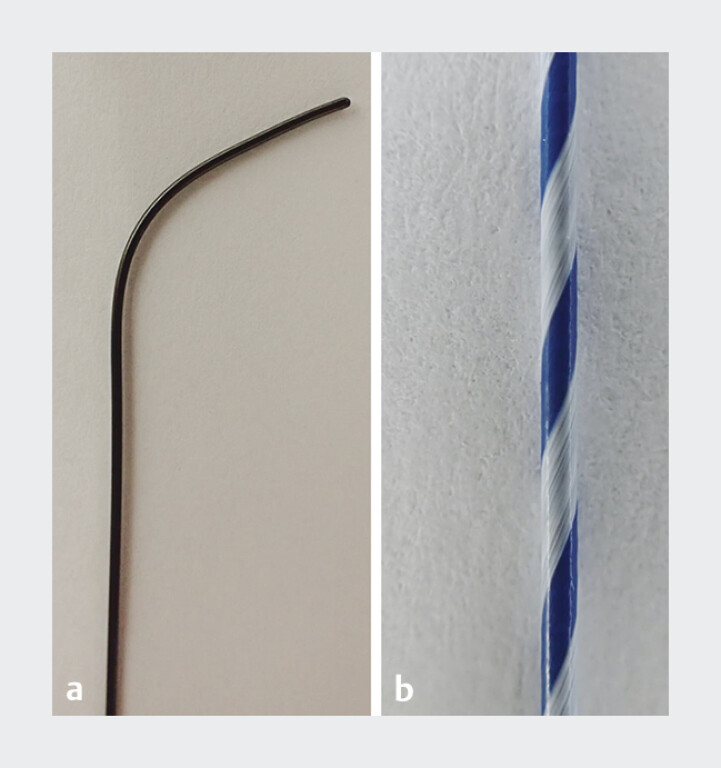
The novel specialized guidewire (DualMaster; Piolax Medical Devices, Kanagawa, Japan) features a 0.025-in angled-tip type, with a significantly larger angle (60°) than conventional guidewires and a longer tip length from the bend to the end (14 mm). **a** The core wire inside the tip exhibits passive flexibility, preventing the loss of tip traction and slipping toward the common bile duct upon initial contact with the right hepatic duct. These features facilitate selective access, even in cases of steep bifurcations. **b** The guidewire shaft has a textured surface, which minimizes friction with other instruments. This provides excellent torqueability and rotational performance, enhancing the high selectivity of the guidewire.

A 60-year-old woman with obstructive jaundice caused by malignant hilar biliary obstruction underwent EUS-HGS with plastic stent placement following ERCP failure. After 2 weeks, a duodenoscope was inserted to introduce a guidewire alongside the HGS stent, followed by removal of the plastic stent. Despite successfully advancing the guidewire through the stricture into the duodenum, attempts to cannulate the right hepatic duct using a double-lumen catheter and a conventional guidewire failed due to the steep bifurcation angle. Therefore, a novel guidewire was introduced. Its enlarged angled tip successfully engaged the right hepatic duct. Through rotational manipulation, the guidewire was advanced into the anterior branch of the right hepatic duct. Ultimately, bilateral stent-in-stent deployment was achieved, with the plastic stent in the HGS fistula being replaced ( Fig. 2 , Video 1 ). The procedure was clinically successful, with no adverse events reported.

**Fig. 2:**
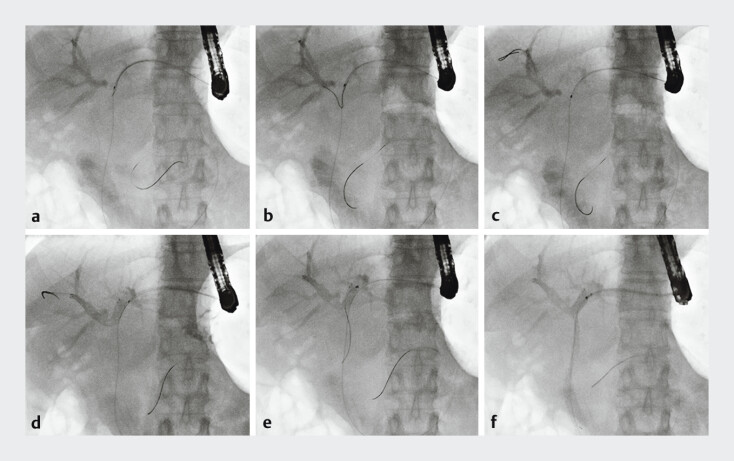
A guidewire was introduced alongside the hepaticogastrostomy stent, followed by removal of the plastic stent. **a** The guidewire was inserted through the stricture into the duodenum, and cannulation of the right hepatic duct was attempted using a double-lumen catheter and the novel guidewire. **b** The enlarged angled tip of the guidewire engaged with the right hepatic duct. **c** Next, rotational manipulation was used to navigate the guidewire into the anterior branch of the right hepatic duct. **d** A metal stent was subsequently deployed, bridging the right and left hepatic ducts. **e** The catheter was reinserted over the guidewire, which was advanced through the stent mesh into the duodenum. **f** Another metal stent was placed antegradely from the duodenum to the left hepatic duct in a stent-in-stent configuration. Finally, a plastic stent was placed across the hepaticgastrostomy fistula.

Bridging deployment into the right hepatic duct via endoscopic ultrasound-guided hepaticogastrostomy was performed using a novel specialized guidewire in a 60-year-old woman with malignant hilar biliary obstruction.Video 1

Endoscopy_UCTN_Code_TTT_1AS_2AH

## Notes

Endoscopy E-Videos https://eref.thieme.de/e-videos
*E-Videos* is an open access online section of the journal *Endoscopy* , reporting on interesting cases and new techniques in gastroenterological endoscopy. All papers include a high-quality video and are published with a Creative Commons CC-BY license. Endoscopy E-Videos qualify for HINARI discounts and waivers and eligibility is automatically checked during the submission process. We grant 100% waivers to articles whose corresponding authors are based in Group A countries and 50% waivers to those who are based in Group B countries as classified by Research4Life (see: https://www.research4life.org/access/eligibility/ ). This section has its own submission website at https://mc.manuscriptcentral.com/e-videos .
